# Group Multimodal Prenatal Care and Postpartum Outcomes

**DOI:** 10.1001/jamanetworkopen.2024.12280

**Published:** 2024-05-21

**Authors:** Lyndsay A. Avalos, Nina Oberman, Lizeth Gomez, Charles P. Quesenberry, Fiona Sinclair, Elaine Kurtovich, Erica P. Gunderson, Monique M. Hedderson, Joanna Stark

**Affiliations:** 1 Division of Research, Kaiser Permanente Northern California, Oakland; 2 Bernard J. Tyson Kaiser Permanente School of Medicine, Pasadena, California; 3 Regional Offices, Kaiser Permanente Northern California, Oakland

## Abstract

**Importance:**

An increasing body of evidence suggests equivalent if not improved postpartum outcomes of in-person group prenatal care compared with individual prenatal care. However, research is needed to evaluate outcomes of group multimodal prenatal care (GMPC), with groups delivered virtually in combination with individual in-person office appointments to collect vital signs and conduct other tests compared with individual multimodal prenatal care (IMPC) delivered through a combination of remotely delivered and in-person visits.

**Objective:**

To compare postpartum outcomes between GMPC and IMPC.

**Design, Setting, and Participants:**

A frequency-matched longitudinal cohort study was conducted at Kaiser Permanente Northern California, an integrated health care delivery system. Participants included 424 individuals who were pregnant (212 GMPC and 212 frequency-matched IMPC controls (matched on gestational age, race and ethnicity, insurance status, and maternal age) receiving prenatal care between August 17, 2020, and April 1, 2021. Participants completed a baseline survey before 14 weeks’ gestation and a follow-up survey between 4 and 8 weeks post partum. Data analysis was performed from January 3, 2022, to March 4, 2024.

**Exposure:**

GMPC vs IMPC.

**Main Outcome Measures:**

Validated instruments were used to ascertain postpartum psychosocial outcomes (stress, depression, anxiety) and perceived quality of prenatal care. Self-reported outcomes included behavioral outcomes (breastfeeding initiation, use of long-acting reversible contraception), satisfaction with prenatal care, and preparation for self and baby care after delivery. Primary analyses included all study participants in the final cohort. Three secondary dose-stratified analyses included individuals who attended at least 1 visit, 5 visits, and 70% of visits. Log-binomial regression and linear regression analyses were conducted.

**Results:**

The final analytic cohort of 390 participants (95.6% follow-up rate of 408 singleton live births) was racially and ethnically diverse: 98 (25.1%) Asian/Pacific Islander, 88 (22.6%) Hispanic, 17 (4.4%) non-Hispanic Black, 161 (41.3%) non-Hispanic White, and 26 (6.7%) multiracial participants; median age was 32 (IQR, 30-35) years. In the primary analysis, after adjustment, GMPC was associated with a 21% decreased risk of perceived stress (adjusted risk ratio [ARR], 0.79; 95% CI, 0.67-0.94) compared with IMPC. Findings were consistent in the dose-stratified analyses. There were no significant differences between GMPC and IMPC for other psychosocial outcomes. While in the primary analyses there was no significant group differences in perceived quality of prenatal care (mean difference [MD], 0.01; 95% CI, −0.12 to 0.15) and feeling prepared to take care of baby at home (ARR, 1.09; 95% CI, 0.96-1.23), the dose-stratified analyses documented higher perceived quality of prenatal care (MD, 0.16; 95% CI, 0.01-0.31) and preparation for taking care of baby at home (ARR, 1.27; 95% CI, 1.13-1.43) for GMPC among those attending 70% of visits. No significant differences were noted in patient overall satisfaction with prenatal care and feeling prepared for taking care of themselves after delivery.

**Conclusions:**

In this cohort study, equivalent and, in some cases, better outcomes were observed for GMPC compared with IMPC. Health care systems implementing multimodal models of care may consider incorporating virtual group prenatal care as a prenatal care option for patients.

## Key Points

**Question:**

Is group prenatal care delivered virtually with individual in-person office visits associated with better postpartum psychosocial, behavioral, and perceived quality of prenatal care outcomes, satisfaction with prenatal care, and preparation for self and baby care compared with individual prenatal care delivered with a combination of remote and in-person visits?

**Findings:**

In this cohort study of 390 participants who were pregnant, group care was associated with equivalent, and in some cases better, outcomes compared with individual care.

**Meaning:**

The findings of this study suggest that health care systems implementing multimodal models of care should consider incorporating virtual group prenatal care as an option for individuals who are pregnant.

## Teaser

This cohort study compares postpartum psychosocial outcomes and perceived quality of prenatal care associated with group prenatal care delivered virtually in combination with in-person office visits vs individual prenatal care delivered through a combination of virtual and in-person office visits.

## Introduction

An increasing body of evidence suggests equivalent if not better perinatal psychosocial (eg, depression, social support)^1,2,3,4,5,6,7,8,9,10^ and behavioral (eg, breastfeeding)^11,12,13,14,15,16^ outcomes and patient satisfaction with care^11^ of in-person group prenatal care compared with in-person individual prenatal care. Group prenatal care brings together 8 to 12 individuals who are pregnant and their partners with a visit schedule and content that follows nationally recognized guidelines with individual medical checks. In contrast to what is provided in individual prenatal care, the group sessions offer an engaging curriculum in a supportive environment that allows for relationship-building, enhances social support among participants, and integrates clinical and behavioral interventions.^17^

The COVID-19 pandemic had a major impact on prenatal health care delivery, requiring a rapid shift to a multimodal model of care. Remotely delivered care, including telephone or video visits, was adopted and integrated into prenatal care to decrease the number of in-person visits and risk of COVID-19 exposure.^18^ However, outcomes of virtual group prenatal care are unknown.

This study investigated whether group multimodal prenatal care (GMPC) (ie, group prenatal care delivered virtually in combination with individual in-person office appointments to collect vital signs and conduct other tests) was associated with better postpartum psychosocial, behavioral, perceived quality of prenatal care, patient satisfaction with prenatal care, and preparation for self and baby care after delivery outcomes compared with individual multimodal prenatal care (IMPC) (ie, individual prenatal care delivered through a combination of remotely delivered and in-person visits).

## Methods

### Study Setting

This cohort study was conducted in Kaiser Permanente Northern California (KPNC), a large integrated health care delivery system that provides medical care to more than 4.6 million members. All 15 regional service centers (with 48 associated office facilities) have obstetrics and gynecology practices. Kaiser Permanente Northern California members are covered by employer-sponsored insurance plans, the insurance exchange, and Medicaid. Coverage is provided for approximately 40% of the northern California population; membership is similar to the population living in the geographic area.^19^ This study was approved by the KPNC Institutional Review Board. Participants provided informed consent and received financial compensations. The Strengthening the Reporting of Observational Studies in Epidemiology (STROBE) reporting guideline reporting for cohort studies was followed.

#### Individual Multimodal Prenatal Care

Kaiser Permanente Northern California individual perinatal care follows the care delivery schedule recommended by the American College of Obstetricians and Gynecologists and includes nine 15-minute individual visits spread across pregnancy, increasing in frequency closer to delivery, and one 15-minute visit at 4 to 10 weeks post partum.^20^ In March 2020, in response to the COVID-19 pandemic, KPNC implemented a multimodal model of prenatal care delivery in which 4 prenatal care visits were offered as remote visits (via video or telephone according to patient preference): 10- to 12-week visit (after completion of the first-trimester office physical examination and ultrasonography examination), 22-week visit, 28-week visit, and 32-week visit.

#### Group Multimodal Prenatal Care

Prior to March 2020, KPNC offered in-person CenteringPregnancy (Centering Healthcare Institute) group prenatal care at 8 medical center service areas throughout the region. After March 2020, KPNC clinics stopped offering in-person groups and adapted CenteringPregnancy to a virtual format. Each clinic built a schedule of 7 to 10 interactive 60- to 90-minute virtual webinars conducted with 8 to 12 individuals who were pregnant and their partners and 10- to 15-minute individual in-person office appointments generally around the same time with their clinician to collect vital signs, weight, and other necessary tests in a schedule that mirrored IMPC. Sessions were facilitative and not didactic, using games to enable engagement, community building, and adult learning. Additionally, each virtual group covered 2 curriculum topics (eTable 1 in Supplement 1) per session and mirrored the curriculum previously offered through in-person group prenatal care.

### Study Design and Population

**Figure 1.**
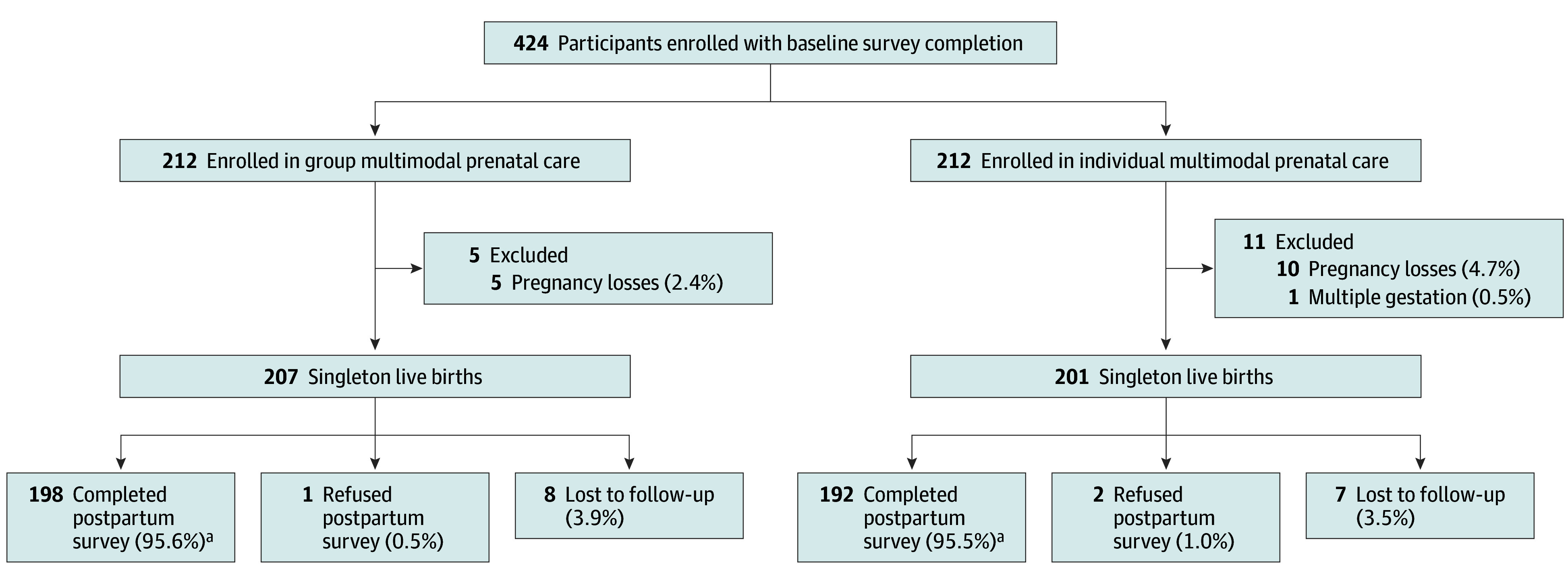
Study Follow-Up Flow Diagram ^a^Includes 3 partially completed.

We conducted a frequency-matched longitudinal cohort study of 424 individuals receiving prenatal care in KPNC (212 participating in GMPC and 212 frequency-matched controls in IMPC) between August 17, 2020, and April 1, 2021 (Figure 1). Controls were matched on gestational age in weeks (between 3 weeks earlier and 1 week after), self-reported race and ethnicity (Hispanic, non-Hispanic Black, non-Hispanic White, Other), insurance status (Medicaid, private/employer sponsored), and maternal age (18-24, 25-30, 31-35, 36-45 years). English-speaking individuals between the ages of 18 and 45 years with singleton pregnancies in their first or early second trimester of gestation and receiving care at 1 of the KPNC prenatal care clinics were eligible for the study. Patients with high-risk pregnancies (ie, chronic hypertension, diabetes) were excluded, as they are not eligible to participate in group prenatal care at many KPNC clinics. After providing informed consent, participants completed a baseline survey and a follow-up survey between 4 and 8 weeks post partum.

Potential GMPC study participants were identified from the electronic health records (EHRs), using the CenteringPregnancy procedure code for a future appointment. Electronic health records databases were used to identify potential IMPC frequency-matched (gestational age, race and ethnicity, insurance status, and maternal age) controls from clinics that did not offer GMPC. With the goal of identifying 1 control per enrolled GMPC participant, up to 15 frequency-matched controls were identified and sent a recruitment email. The first IMPC individual for each GMPC participant who responded with interest received a phone call to assess eligibility. Given that people who choose to participate in group prenatal care may be different from those who choose not to, after the research assistant described CenteringPregnancy, only individuals who responded that they would have participated in GMPC if given the option were recruited. If they were ineligible or refused, another potential participant who received the recruitment email was called. This continued until an IMPC control was enrolled for each GMPC participant. If an IMPC control was not identified from the pool of potential participants, up to 15 more were identified from the EHRs to contact.

Internal encounter and clinician codes in the EHRs were used to identify data on completion of virtual group prenatal and postpartum sessions (GMPC) and virtual, telephone or in-person individual prenatal and postpartum care visits (IMPC).

## Outcomes

Outcomes were ascertained from the postpartum survey. Depression was assessed using the validated 8-item, Patient Health Questionnaire screener.^21,22^ Scores of 10 to 24 (range, 0-24) were categorized as clinically significant depressive symptoms.^22^ Anxiety was measured using the validated 7-item Generalized Anxiety Disorder Scale.^23,24,25,26^ Scores of 10 to 21 (range, 0-21) were categorized as clinically significant anxiety symptoms.^23^ Perceived stress was evaluated using the widely validated Perceived Stress Scale.^8,27,28^ Scores of 14 or greater (range, 0 to 40) were categorized as moderate to severe perceived stress. Sleep quality was assessed via the validated 19-item Pittsburgh Sleep Quality Index),^29^ with higher scores (range, 0-21) indicating poorer sleep quality. Social support was measured using the Medical Outcomes Study Social Support Survey.^30^ Higher scores (range, 1-5) reflect higher levels of social support.

Behavioral outcomes included breastfeeding initiation (yes or no) and long-acting reversible contraception (LARC) intention, defined as intention to use an implant (eg, etonogestrel) or intrauterine device (eg, ParaGard or Mirena) in the next 5 months.

Perceived quality of prenatal care was evaluated using the validated 46-item Quality of Prenatal Care Questionnaire^31^ with 5-item Likert responses (range, 1-5). Higher scores indicate greater perceived prenatal care quality. Prenatal care satisfaction and preparation for self and baby care were evaluated using a 5-item Likert scale for the following statements: (1) overall, I was satisfied with my prenatal care, (2) my prenatal care prepared me for taking care of myself at home, and (3) my prenatal care prepared me for taking care of my baby at home. Responses were categorized as yes (strongly agree or agree) vs no (neither agree nor disagree, disagree, or strongly disagree).

Secondary outcomes were the subscales for sleep quality, social support, and perceived quality of prenatal care surveys (subscales are provided in the eAppendix in Supplement 1).

Covariates ascertained from EHRs at baseline and used in match selection were maternal age (years) at pregnancy onset (18-24, 25-30, 31-35, and 36-45), gestational age in weeks on date of identification, insurance status (Medicaid, private/employer sponsored), and self-reported race (EHR categories included Asian, Black, Hawaiian/Pacific Islander, Native American, White, Multiracial, unknown) and ethnicity (Hispanic or non-Hispanic). For match selection, patient race and ethnicity were categorized as non-Hispanic Black, Hispanic, non-Hispanic White, and non-Hispanic Other (Asian, Hawaiian/Pacific Islander, Native American, Multiracial, or unknown).

Additional covariates ascertained from responses on baseline surveys were annual household income (less than or equal to $99 000 per year, $100 000 to $149 000 per year, $150 000 and greater per year), parity (0, ≥1) and race (American Indian/Alaska Native, Asian, Native Hawaiian or Other Pacific Islander, Black or African American, White), and ethnicity (Hispanic/Latino or not Hispanic/Latino). The race and ethnicity variable included in the statistical analysis was sourced from survey data and supplemented by EHR data for nonresponders and categorized as Asian/Pacific Islander, non-Hispanic Black, Hispanic, non-Hispanic White, or multiracial.

We originally planned for 125 participants in each of the 2 study groups but were successful in recruiting additional participants. Analyses are based on 198 individuals in the GMPC group and 192 in the IMPC group, and we therefore present minimum detectable results associated with our final sample sizes. With these sample sizes, the minimum detectable risk ratios (GMPC vs IMPC) are 0.42 for anxiety, 0.50 for depression, 0.80 for stress, 1.11 for breastfeeding, and 1.37 for LARC (power = 0.80; α = .05; 2-sided test; expected proportions in IMPC group: 0.15 for anxiety,^32^ 0.20 for depression,^33^ 0.67 for stress,^34^ 0.83 for breastfeeding,^35^ and 0.38 for LARC^36^). The minimum detectable between-group difference in means is 0.28 SD units.

### Statistical Analysis

Data analysis was performed from January 3, 2022, to March 4, 2024. Our primary analysis of between-group differences in outcomes of interest included all participants who completed the postpartum survey regardless of the number (dose) of prenatal care visits attended (ie, group prenatal care visits for participants in the GMPC group and in-person or virtual individual visits for those in the IMPC group). We conducted 3 secondary analyses stratified on varying levels of prenatal care use (dose-stratified analyses); we compared outcomes of GMPC vs IMPC participants who completed at least 1 prenatal care visit (DS1), 5 prenatal care visits (DS5), and 70% of prenatal care visits (DS70%) The 5-visit threshold was based on previous research defining this cutoff as a marker of adequate exposure to in-person group prenatal care.^14,37,38,39,40,41,42^ The 70% threshold was conducted to address variation in the number of virtual GMPC group sessions offered by KPNC clinics, which ranged from 7 to 10. For IMPC participants, the total recommended number of prenatal care visits after study enrollment was 8.

Descriptive statistics were calculated overall and by study group. Medians and IQRs were calculated to describe the distribution of continuous variables, and frequency and percent were calculated to describe categorical variables.

Log-binomial regression (ie, generalized linear model with binomial distribution and log link) was used to estimate risk ratios (RRs) and 95% CIs for binary outcomes (ie, perceived stress, depression, anxiety, breastfeeding, LARC intention, satisfaction with prenatal care, and preparation for self and baby care). Linear regression was used to estimate the between-group mean differences (MDs) for continuous outcome scales (ie, sleep quality, social support, and perceived quality of prenatal care). Estimates from regression analyses are interpreted as marginal effects/population averages. Variables used for frequency matching of the 2 groups were included in all regression models for increased precision in estimation of between-group differences; however, because no IMPC participants had Medicaid insurance at baseline, Medicaid status was not included in the models. All models were adjusted for additional a priori–selected covariates: baseline score on the outcome scale of interest (categorical for binary outcomes, continuous for continuous outcomes), annual household income (categorical), parity (categorical), and gestational age in weeks at baseline survey completion (continuous).

Analyses were conducted in R, version 4.0.2 (R Foundation for Statistical Computing). Two-sided *P* values <.05 were considered statistically significant.

## Results

### Study Recruitment and Sample Characteristics

A total of 551 potentially eligible GMPC participants were identified in the EHRs and contacted for recruitment. Of these, 212 enrolled in the study (more details in in the eMethods in Supplement 1). Overall, 207 GMPC participants had a singleton live birth and 198 individuals (95.6%) completed the follow-up survey (Figure 1).

A total of 2955 IMPC individuals were identified as potential matches for the 212 enrolled GMPC participants. Of these, 594 were screened for eligibility and 212 enrolled in the study (more details available in the eMethods in Supplement 1). Overall, 201 experienced a singleton live birth and 192 individuals (95.5%) completed a postpartum follow-up survey (Figure 1).

The analytic cohort of 390 pregnant individuals was racially and ethnically diverse (98 [25.1%] Asian/Pacific Islander, 88 [22.6%] Hispanic, 17 [4.4%] non-Hispanic Black, 161 [41.3%] non-Hispanic White, 26 [6.7%] multiracial), with a median age of 32 (IQR, 30-35) years, and the median gestational age at study entry was 13 (IQR, 11-15) weeks (Table). Baseline characteristics were balanced between GMPC and IMPC groups with a few exceptions; GMPC participants were more likely to have a higher household income and higher educational level, and less likely to have older children (χ^2^ *P* < .001). There were no significant differences in baseline characteristics between those lost to follow-up/refused (n = 18) and those who completed the postpartum survey (n = 390) (eTable 2 in Supplement 1). Postpartum surveys were completed at a median of 6 (IQR, 5-8) weeks post partum (GMPC: median, 7 [IQR, 5-9] weeks, IMPC: median, 5 [IQR, 4-6] weeks).

**Table.** Participant Baseline Characteristics

Characteristic	Patients, No. (%)								
	Overall (N = 390)	Primary analysis		DS1 visit analysis		DS5 visits analysis		DS70% visits analysis	
		IMPC (n = 192)	GMPC (n = 198)	IMPC (n = 188)	GMPC (n = 177)	IMPC (n = 171)	GMPC (n = 115)	IMPC (n = 164)	GMPC (n = 99)
Race and ethnicity									
Asian/Pacific Islander	98 (25.1)	47 (24.5)	51 (25.8)	45 (23.9)	44 (24.9)	41 (24.0)	22 (19.1)	39 (23.8)	20 (20.2)
Hispanic	88 (22.6)	45 (23.4)	43 (21.7)	44 (23.4)	39 (22.0)	38 (22.2)	19 (16.5)	37 (22.6)	18 (18.2)
Non-Hispanic Black	17 (4.4)	8 (4.2)	9 (4.5)	8 (4.3)	8 (4.5)	7 (4.1)	5 (4.3)	7 (4.3)	5 (5.1)
Non-Hispanic White	161 (41.3)	78 (40.6)	83 (41.9)	77 (41.0)	77 (43.5)	72 (42.1)	61 (53.0)	69 (42.1)	50 (50.5)
Multiracial	26 (6.7)	14 (7.3)	12 (6.1)	14 (7.4)	9 (5.1)	13 (7.6)	8 (7.0)	12 (7.3)	6 (6.1)
Age at pregnancy onset, median (IQR), y	32 (30-35)	32 (30-35)	32 (30-35)	32 (30-35)	33 (30-35)	32 (30-35)	33 (30-35)	32 (30-35)	33 (30-35)
Medicaid insurance at baseline	3 (0.8)	0	3 (1.5)	0	1 (0.6)	0	0	0	0
Annual household income, $									
≤99 000	106 (27.2)	70 (36.5)	36 (18.2)	68 (36.2)	29 (16.4)	60 (35.1)	14 (12.2)	58 (35.4)	10 (10.1)
100 000-149 000	90 (23.1)	48 (25.0)	42 (21.2)	47 (25.0)	35 (19.8)	44 (25.7)	21 (18.3)	43 (26.2)	18 (18.2)
≥149 999	173 (44.4)	68 (35.4)	105 (53.0)	67 (35.6)	100 (56.5)	61 (35.7)	74 (64.3)	57 (34.8)	66 (66.7)
Not reported	21 (5.4)	6 (3.1)	15 (7.6)	6 (3.2)	13 (7.3)	6 (3.5)	6 (5.2)	6 (3.7)	5 (5.1)
Highest level of education									
High school or less	39 (10.0)	28 (14.6)	11 (5.6)	28 (14.9)	7 (4.0)	26 (15.2)	3 (2.6)	26 (15.9)	3 (3.0)
Any college	176 (45.1)	91 (47.4)	85 (42.9)	88 (46.8)	72 (40.7)	78 (45.6)	38 (33.0)	75 (45.7)	34 (34.3)
Any postgraduate	173 (44.4)	73 (38.0)	100 (50.5)	72 (38.3)	96 (54.2)	67 (39.2)	73 (63.5)	63 (38.4)	62 (62.6)
Not reported	2 (0.5)	0	2 (1.0)	0	2 (1.1)	0	1 (0.9)	0	0
Parity									
Nulliparous	261 (66.9)	100 (52.1)	161 (81.3)	98 (52.1)	147 (83.1)	91 (53.2)	96 (83.5)	87 (53.0)	81 (81.8)
Multiparous	129 (33.1)	92 (47.9)	37 (18.7)	90 (47.9)	30 (16.9)	80 (46.8)	19 (16.5)	77 (47.0)	18 (18.2)
Gestational age at baseline survey completion, median (IQR), wk	13 (11-14)	13 (11-15)	13 (11-14)	13 (11-15)	13 (10-14)	12 (11-15)	12 (10-14)	12 (11-15)	12 (10-14)
Baseline PHQ-8 score category									
0-4: No to minimal depression	214 (54.9)	105 (54.7)	109 (55.1)	104 (55.3)	96 (54.2)	95 (55.6)	59 (51.3)	90 (54.9)	50 (50.5)
5-9: Mild depression	127 (32.6)	58 (30.2)	69 (34.8)	56 (29.8)	63 (35.6)	50 (29.2)	41 (35.7)	50 (30.5)	34 (34.3)
10-24: Moderate to severe depression	49 (12.6)	29 (15.1)	20 (10.1)	28 (14.9)	18 (10.2)	26 (15.2)	15 (13.0)	24 (14.6)	15 (15.2)
Baseline PSS-10 score category									
0-13: Low stress	190 (48.7)	88 (45.8)	102 (51.5)	87 (46.3)	94 (53.1)	81 (47.4)	62 (53.9)	77 (47.0)	53 (53.5)
14-26: Moderate stress	189 (48.5)	96 (50.0)	93 (47.0)	93 (49.5)	80 (45.2)	83 (48.5)	51 (44.3)	81 (49.4)	44 (44.4)
≥27: High perceived stress	11 (2.8)	8 (4.2)	3 (1.5)	8 (4.3)	3 (1.7)	7 (4.1)	2 (1.7)	6 (3.7)	2 (2.0)
Baseline GAD-7 score category									
0-4: Normal anxiety	265 (67.9)	124 (64.6)	141 (71.2)	120 (63.8)	129 (72.9)	109 (63.7)	82 (71.3)	105 (64.0)	68 (68.7)
5-9: Mild anxiety	94 (24.1)	47 (24.5)	47 (23.7)	47 (25.0)	38 (21.5)	44 (25.7)	26 (22.6)	43 (26.2)	24 (24.2)
10-21: Moderate to severe anxiety	31 (7.9)	21 (10.9)	10 (5.1)	21 (11.2)	10 (5.6)	18 (10.5)	7 (6.1)	16 (9.8)	7 (7.1)
Baseline MOS-SSS score, mean (SD)	4.5 (0.6)	4.4 (0.7)	4.5 (0.6)	4.4 (0.7)	4.5 (0.6)	4.4 (0.7)	4.6 (0.6)	4.4 (0.7)	4.5 (0.6)
Baseline PSQI score, mean (SD)	6.0 (3.3)	6.1 (3.5)	5.9 (3.2)	6.1 (3.4)	5.8 (3.2)	6.1 (3.4)	5.7 (3.0)	6.1 (3.4)	5.7 (3.1)

Abbreviations: DS1, dose-stratified, at least 1 visit required; DS5, dose-stratified, at least 5 visits required; DS70%, dose-stratified, at least 70% of visits required; GAD-7, 7-item Generalized Anxiety Disorder Scale; GMPC, group multimodal prenatal care; IMPC, individual multimodal prenatal care; MOS-SSS, Medical Outcomes Study Social Support Survey; PHQ-8, 8-Item Patient Health Questionnaire depression scale; PSS-10, 10-Item Perceived Stress Scale; PSQI, Pittsburgh Sleep Quality Index.

Participants in the GMPC study group attended a median of 68.4% (IQR, 30.0%-85.7%) of virtual group visits; 99 (50.0%) attended at least 70% of the virtual group visits, 115 (58.1%) attended at least 5 virtual groups, and 177 (89.4%) attended at least 1 virtual group. Participants in the IMPC study group attended a median of 7 (IQR, 6-8) prenatal visits, of which a median of 14.3% (IQR, 0%-28.6%) were virtual. Of the IMPC participants, 188 (97.9%) attended at least 1 prenatal visit, 180 (93.8%) attended at least 5 visits, and 164 (85.4%) attended at least 70% (at least 6 of 8) of visits.

### Primary Analysis

**Figure 2.**
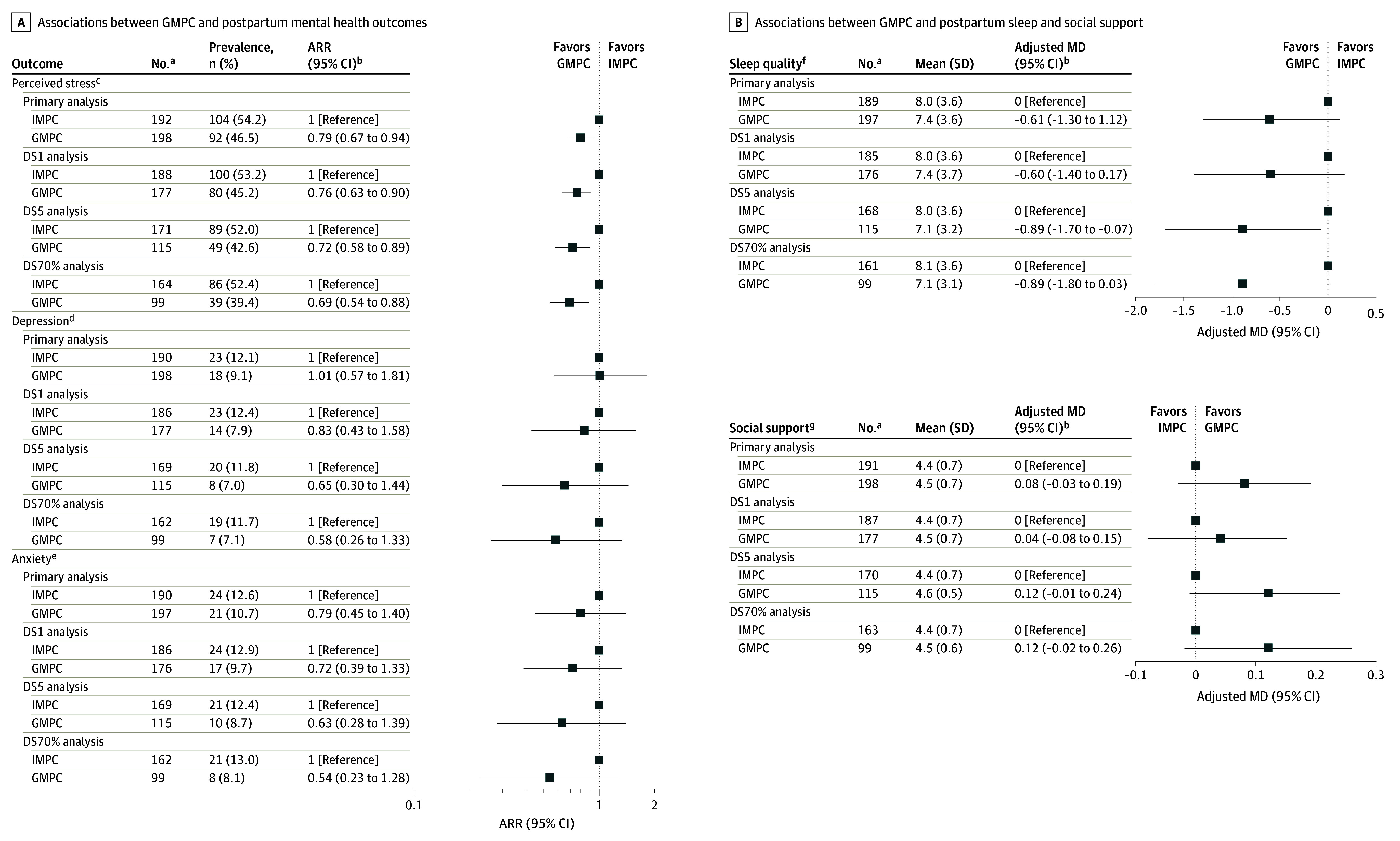
Associations Between Group Multimodal Prenatal Care (GMPC) and Postpartum Psychosocial and Behavioral Outcomes and Perceived Quality of Prenatal Care Associations between GMPC and postpartum mental health outcomes (A) and postpartum sleep and social support (B). ARR indicates adjusted relative risk; DS1, dose-stratified, at least 1 visit required; DS5, dose-stratified, at least 5 visits required; DS70%, dose-stratified, at least 70% of visits required; IMPC, individual multimodal prenatal care; and MD, mean difference. ^a^Difference in sample sizes due to partial completions of postpartum survey. ^b^Adjusted for race and ethnicity, age at pregnancy onset, annual household income level, parity, gestational age at baseline survey completion, and baseline score category on outcome scale. ^c^Score of 14 or greater on the Perceived Stress Scale. This indicates moderate to severe perceived stress. ^d^Score of 10 or greater on the Patient Health Questionnaire depression scale.This indicates clinically significant depressive symptoms. ^e^Score of 10 or greater on the Generalized Anxiety Disorder 7-item scale. This indicates clinically significant depressive symptoms ^f^Pittsburgh Sleep Quality Index Score. Range: 0 (better) to 21 (worse). ^g^Medical Outcomes Study Social Support Survey score. Range: 1 (low) to 5 (high).

**Figure 3.**
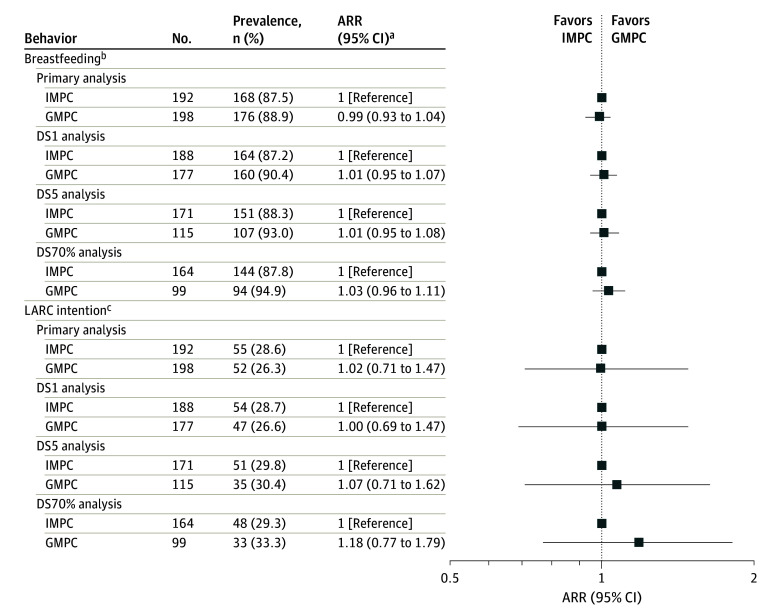
Associations Between Group Multimodal Prenatal Care (GMPC) and Breastfeeding and Postpartum Long-Acting Reversible Contraceptive (LARC) Intention ARR indicates adjusted relative risk; DS1, dose-stratified, at least 1 visit required; DS5, dose-stratified, at least 5 visits required; DS70%, dose-stratified, at least 70% of visits required; and IMPC, individual multimodal prenatal care. ^a^Adjusted for race and ethnicity, age at pregnancy onset, annual household income level, parity, and gestational age at baseline survey completion. ^b^Any self-reported breastfeeding since birth. ^c^Self-reported intention to use an LARC method in the postpartum period.

**Figure 4.**
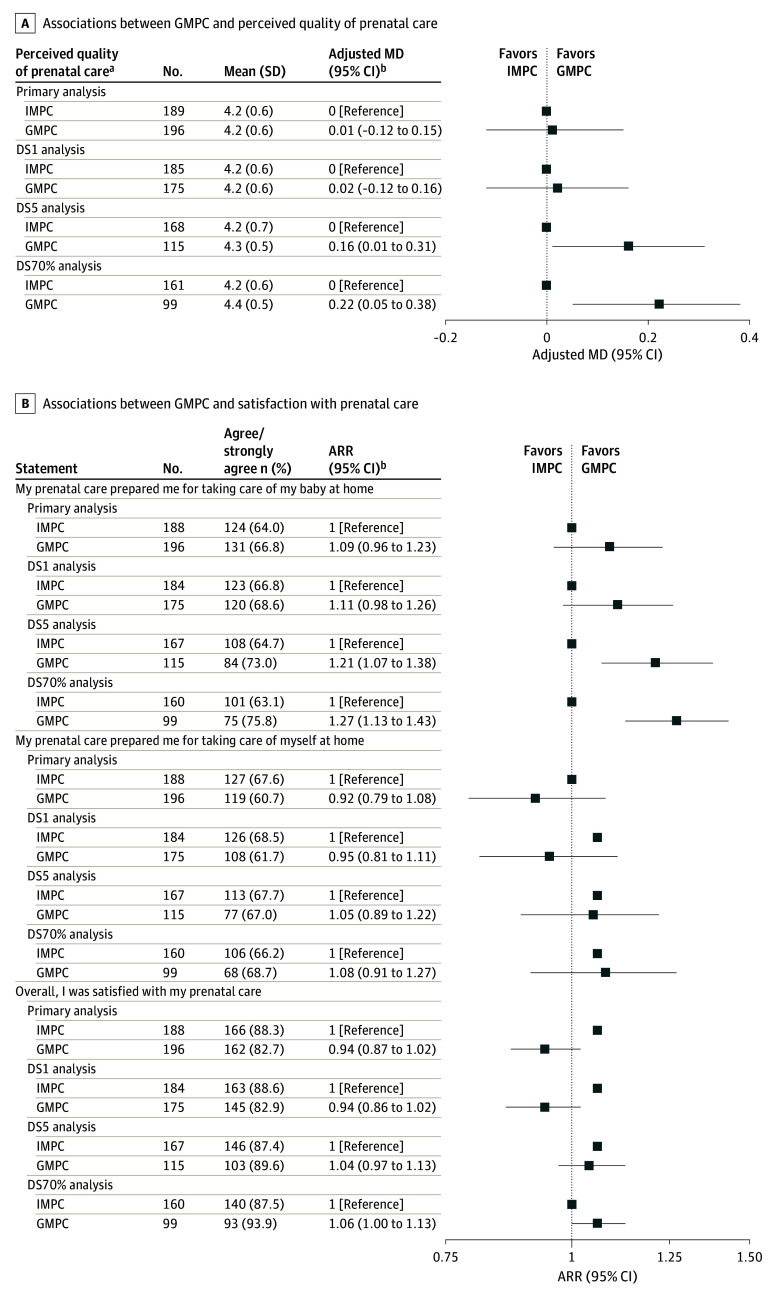
Associations Between Group Multimodal Prenatal Care (GMPC) and Perceived Quality of and Satisfaction With Prenatal Care Outcomes assessed at postpartum survey for association between GMPC and perceived quality of care (A) and satisfaction with prenatal care (B). ARR indicates adjusted relative risk; DS1, dose-stratified, at least 1 visit required; DS5, dose-stratified, at least 5 visits required; DS70%, dose-stratified, at least 70% of visits required; and IMPC, individual multimodal prenatal care. ^a^Quality of Prenatal Care Questionnaire assessed at postpartum survey. Total 46-item mean score, range: 1 (worse) to 5 (better). ^b^Adjusted for race and ethnicity, age at pregnancy onset, annual household income level, parity, and gestational age at baseline survey completion.

After adjustment for potential confounders, GMPC was associated with a 21% decreased risk of perceived stress (adjusted RR [ARR], 0.79; 95% CI, 0.67-0.94) compared with IMPC (Figure 2A; eTable 3 in Supplement 1). There were no significant differences between GMPC and IMPC for any of the other psychosocial outcomes, including depression (ARR, 1.01; 95% CI, 0.57-1.81), anxiety (ARR, 0.79; 95% CI, 0.45-1.40), sleep quality (MD, −0.61; 95% CI, −1.3 to 0.12), and greater social support (MD, 0.08; 95% CI, −0.03 to 0.19) compared with IMPC (Figures 2A and B; eTable 3 and eTable 4 in Supplement 1). No significant differences were observed between GMPC and IMPC regarding breastfeeding (ARR, 0.99; 95% CI, 0.93-1.04) and LARC intention (ARR, 1.02; 95% CI, 0.71-1.47) (Figure 3; eTable 5 in Supplement 1). No significant differences were observed between GMPC and IMPC for perceived quality of prenatal care (MD, 0.01; 95% CI, −0.12 to 0.15) Figure 4A), patient satisfaction with prenatal care (ARR, 0.94; 95% CI, 0.87-1.02), or feeling prepared for taking care of themselves (ARR, 0.92; 95% CI, 0.79-1.08) or their baby (ARR, 1.09; 95% CI, 0.96-1.23) at home (Figure 4B).

Greater sleep duration was noted with GMPC compared with IMPC after adjustment (MD, −0.19; 95% CI, −0.35 to −0.03), but no other significant differences were noted for any other subscales (eTable 7 in Supplement 1).

### Dose-Stratified Analyses

Findings from the DS1 analysis were similar to the primary analysis of all recruited study participants. However, in the DS5 and DS70% models, better outcomes for GMPC were documented for overall perceived quality of care (DS5: MD, 0.16; 95% CI, 0.01-0.31) (Figure 4A; eTable 6 in Supplement 1) and preparation for baby care (DS70%: RR, 1.27; 95% CI, 1.13-1.43) (Figure 4B).

An association with better outcomes was also observed with GMPC in the DS5 and DS70% analyses after adjusting for potential confounders for several of the subscales: sleep (sleep latency [DS5 MD, −0.27; 95% CI, −0.54 to 0.01]), social support (positive social interaction [DS5 MD, 0.17; 95% CI, 0.01 to 0.33]), and perceived quality of prenatal care (higher anticipatory guidance [DS5 MD, 0.31; 95% CI, 0.13-0.49], availability [DS5 MD, 0.22; 95% CI, 0.03-0.41], and for the DS70% only, sufficient time [DS70% MD, 0.24; 95% CI, 0.02-0.45]) (eTable 4 in the Supplement).

## Discussion

In this cohort study comparing group and individual multimodal prenatal care models, findings suggest either better outcomes for GMPC or equivalent outcomes between the 2 models of care. Better perceived stress outcomes for GMPC were noted regardless of the analytic model, which is also of significance given that this study was conducted during the COVID-19 pandemic, a highly stressful period. Although there were no statistically significant differences for any of the other psychosocial, behavioral, perceived quality of prenatal care, and prenatal care satisfaction outcomes or preparedness for taking care of self or baby, the findings reflect better outcomes for GMPC. In the analyses stratified by prenatal care use levels, GMPC was associated with higher perceived quality of prenatal care overall, greater preparation for taking care of the baby at home and higher scores on several subscales, including anticipatory guidance, availability, and sufficient time. Better sleep latency and social interaction subscale scores were also observed for GMPC in the dose-stratified analyses. Findings from the dose-stratified analyses suggest greater benefits for participants with higher GMPC engagement. To our knowledge, this is the first study that has investigated group prenatal care delivered in a multimodal format with all group visits delivered virtually.

Research on multimodal models of prenatal care delivery is sparse; thus, our findings can only be compared with studies of in-person group prenatal care compared with individual care that have similarly documented no statistically significant differences in postpartum depression and anxiety outcomes.^1,2,3,7,8,10^ However, our results contrast with previous studies that failed to find a difference in perceived stress for group compared with individual prenatal care.^7,8,10^ We observed improvement in sleep latency with greater GMPC engagement, which is somewhat similar to other research documenting improved insomnia among women who were overweight or obese in group prenatal care.^43^ Research has been inconsistent with regards to social support outcomes, with some investigations reporting greater social support associated with group prenatal care and others reporting no statistically significant differences.^4,5,6^ Our study contrasts with others that have reported higher rates of breastfeeding for group prenatal care participants.^11,12,13^ We had higher reported rates of breastfeeding initiation overall compared with other studies, and we also investigated initiation as opposed to continuation, which may explain these differences. Despite many studies reporting greater LARC uptake for participants in group prenatal care,^12,13,14,15,16^ we did not find any statistically significant differences in LARC intention. The difference in findings may be due to measuring LARC intention rather than uptake, and also capturing this intention soon after birth (approximately 7 weeks post partum). In addition, similar to one other study, we identified better perceived quality of prenatal care among participants in group prenatal care.^11^

This study provides preliminary evidence of equivalent if not better postpartum outcomes for a multimodal care delivery model with virtual group prenatal care compared with individual prenatal care. A recent study conducted in KPNC documented similar maternal and infant health and health care outcomes for individual in-person prenatal care compared with a multimodal model of prenatal care.^18^ Findings from our study can support health care systems and clinicians as they evaluate and redesign prenatal health care delivery while we continue to transition out of the COVID-19 pandemic.^44^ Specifically, for health care systems implementing multimodal models of care, group prenatal care offered in a virtual format can provide an alternative to in-person group prenatal care.

This study did not compare virtual group prenatal care with in-person group prenatal care, signifying an area for future research. Future research should also investigate associations between GMPC and additional clinical and perinatal outcomes.

### Strengths and Limitations

This study has important strengths. Previous observational studies comparing outcomes of group vs individual prenatal care have used claims or EHR data without knowledge of whether patients receiving individual prenatal care were offered the choice of group prenatal care.^12,13,14,16,39,45^ Individuals who are pregnant and choose to participate in group prenatal care may differ from those who choose individual prenatal care. Previous randomized clinical trials are also limited in their generalizability given that people who agree to participate and be randomized may be different from the general population. This study was designed to address those limitations by selecting controls who would have chosen to participate in GMPC if they were given the choice. Kaiser Permanente Northern California has been partnering with Centering Healthcare Institute since 2011, and clinicians have extensive experience in providing CenteringPregnancy, indicating high fidelity to the model. The frequency-matched study design balanced the groups on certain salient characteristics. We had a 95% follow-up rate in both groups of the study, decreasing the potential of selection bias. Data from EHRs were used to track use of prenatal care to measure adherence. Additionally, the baseline survey captured covariates not traditionally available in the EHRs. Finally, validated questionnaires were used to evaluate outcomes, decreasing the potential for misclassification.

This study has a few limitations. Despite the frequency-matched design, the groups differed on several characteristics. We addressed this through adjustment in multivariable analyses. This study is not generalizable to people who would not choose GMPC if given the option, persons with a high-risk pregnancy (ie, chronic hypertension, diabetes), or without insurance. The detectable effect estimates for anxiety, depression, and LARC were large. In addition, exclusion of participants from the dose-stratified analyses may have been differential on unmeasured factors associated with outcomes.

## Conclusions

The findings of this cohort study suggest equivalent, and in some cases better, postpartum psychosocial and behavioral outcomes, perceived quality of prenatal care, and prenatal care satisfaction for GMPC compared with IMPC. As health care systems implement multimodal models of care, consideration should be given to integrating virtual group prenatal care as an option for patients.

## Notes

Supplement 1. **eTable 1.** CenteringPregnancy Topics **eTable 2.** Baseline Characteristics for Participants Who Completed the Postpartum Survey and Those Who Were Lost to Follow-up, n=408 **eTable 3.** Associations Between GMPC and Postpartum Psychosocial (Mental Health) Outcomes **eTable 4.** Associations Between GMPC and Postpartum Psychosocial (Sleep and Social Support) Outcomes **eTable 5.** Associations Between GMPC and Postpartum Behavioral (Breastfeeding and LARC Intention) **eTable 6.** Associations Between GMPC and Perceived Quality of Prenatal Care **eTable 7.** Associations of GMPC with Postpartum Sleep, Social Support, and Perceived Quality of Prenatal Care Subscales **eMethods.** Additional Details on Identification and Recruitment of the Study Sample. **eAppendix.** The Quality of Prenatal Care Questionnaire Subscales

Supplement 2.
Data Sharing Statement
