# Supplementary material for: Group Multimodal Prenatal Care and Postpartum Outcomes
Source: JAMA Netw Open. 2024 May 21;7(5):e2412280. doi: 10.1001/jamanetworkopen.2024.12280 (PMC11109777; doi:10.1001/jamanetworkopen.2024.12280)
Supplement: Supplement 1. — eTable 1. CenteringPregnancy Topics eTable 2. Baseline Characteristics for Participants Who Completed the Postpartum Survey and Those Who Were Lost to Follow-up, n=408 eTable 3. Associations Between GMPC and Postpartum Psychosocial (Mental Health) Outcomes eTable 4. Associations Between GMPC and Postpartum Psychosocial (Sleep and Social Support) Outcomes eTable 5. Associations Between GMPC and Postpartum Behavioral (Breastfeeding and LARC Intention) eTable 6. Associations Between GMPC and Perceived Quality of Prenatal Care eTable 7. Associations of GMPC with Postpartum Sleep, Social Support, and Perceived Quality of Prenatal Care Subscales eMethods. Additional Details on Identification and Recruitment of the Study Sample. eAppendix. The Quality of Prenatal Care Questionnaire Subscales [file jamanetwopen-e2412280-s001.pdf]

Supplementary Online Content

Avalos LA, Oberman N, Gomez L, et al. Group multimodal prenatal care and postpartum outcomes. *JAMA Netw Open*. 2024;7(5):e2412280.
doi:10.1001/jamanetworkopen.2024.12280

eTable 1. CenteringPregnancy Topics

eTable 2. Baseline Characteristics for Participants Who Completed the Postpartum Survey and Those Who Were Lost to Follow-up, n=408

eTable 3. Associations Between GMPC and Postpartum Psychosocial (Mental Health) Outcomes

eTable 4. Associations Between GMPC and Postpartum Psychosocial (Sleep and Social Support) Outcomes

eTable 5. Associations Between GMPC and Postpartum Behavioral (Breastfeeding and LARC Intention)

eTable 6. Associations Between GMPC and Perceived Quality of Prenatal Care

eTable 7. Associations of GMPC with Postpartum Sleep, Social Support, and Perceived Quality of Prenatal Care Subscales

eMethods. Additional Details on Identification and Recruitment of the Study Sample.

eAppendix. The Quality of Prenatal Care Questionnaire Subscales

This supplementary material has been provided by the authors to give readers additional information about their work.

© 2024 Avalos LA et al. *JAMA Network Open.*

eTable 1. CenteringPregnancy Topics*

Common discomforts
Nutrition
Back care
Healthy relationships
"The Family I Want to Have"
Family responsibilities
Preterm labor
The physiology of labor
Coping with labor
Breastfeeding challenges
Managing sleep deprivation
Contraception
Newborn care & expectations
Mood challenges
Postpartum body changes
Building resiliency
Sharing birth stories

*This list represents a general list of topics covered during CenteringPregnancy, but the list is not exhaustive.

eTable 2. Baseline Characteristics for Participants who Completed the Postpartum Survey and those who were Lost to Follow-up, n=408

	**Completed Postpartum Survey**, N = 390	**Lost to Follow-up/ Refused**, N = 18
Group		
IMPC	192 (49%)	9 (50%)
GMPC	198 (51%)	9 (50%)
Race/ethnicity, n (%)		
Non-Hispanic White	161 (41%)	7 (39%)
Asian/Pacific Islander	98 (25%)	3 (17%)
Hispanic	88 (23%)	5 (28%)
Multiracial	26 (7%)	0 (0%)
Non-Hispanic Black	17 (4%)	3 (17%)
Age at pregnancy onset, mean (SD)	32.3 (4.1)	31.4 (4.1)
Medicaid insurance at baseline, n (%)	3 (1%)	1 (6%)
Annual household income, n (%)		
Less than $99,000 per year	106 (27%)	4 (22%)
$100,000 to $149,000 per year	90 (23%)	4 (22%)
$150,000 and greater per year	173 (44%)	8 (44%)
Not reported	21 (5%)	2 (11%)
Highest level of education, n (%)		
High school or less	39 (10%)	3 (17%)
Any college	176 (45%)	4 (22%)
Any post-graduate	173 (44%)	11 (61%)
Not reported	2 (1%)	0 (0%)
Parity, n (%)		
Nulliparous	261 (67%)	12 (67%)
Multiparous	129 (33%)	6 (33%)
Gestational age at baseline survey completion, mean (SD)	12.6 (2.8)	12.2 (2.5)
Baseline PHQ-8 score category, n (%)		
0-4: No to minimal depression	214 (55%)	8 (44%)
5-9: Mild depression	127 (33%)	8 (44%)
10-24: Moderate to severe depression	49 (13%)	2 (11%)
Baseline PSS-10 score category, n (%)		
0-13: Low stress	190 (49%)	12 (67%)
14-26: Moderate stress	189 (48%)	5 (28%)
27+: High perceived stress	11 (3%)	1 (6%)
Baseline GAD-7 score category, n (%)		
0-4: Normal anxiety	265 (68%)	12 (67%)
5-9: Mild anxiety	94 (24%)	5 (28%)
10-21: Moderate to severe anxiety	31 (8%)	1 (6%)
Baseline MOS-SSS score, mean (SD)	4.5 (0.6)	4.3 (0.7)
Baseline PSQI score, mean (SD)	6.0 (3.3)	5.6 (2.8)

© 2024 Avalos LA et al. *JAMA Network Open.*

eTable 3. Associations Between GMPC and Postpartum Psychosocial (Mental Health) Outcomes

	ITT		PC1		PC5		PC70%	
**Outcome**	cRR (95% CI)	aRR[1] (95% CI)	cRR (95% CI)	aRR[1] (95% CI)	cRR (95% CI)	aRR[1] (95% CI)	cRR (95% CI)	aRR[1] (95% CI)
**Perceived Stress[2]**								
IMPC	Ref	Ref	Ref	Ref	Ref	Ref	Ref	Ref
GMPC	0.86 (0.70, 1.05)	**0.79 (0.67, 0.94)**	0.85 (0.69, 1.05)	**0.76 (0.63, 0.90)**	0.82 (0.63, 1.05)	**0.72 (0.58, 0.89)**	**0.75 (0.56, 0.99)**	**0.69 (0.54, 0.88)**
**Depression[3]**								
IMPC	Ref	Ref	Ref	Ref	Ref	Ref	Ref	Ref
GMPC	0.75 (0.41, 1.34)	1.01 (0.57, 1.81)	0.64 (0.33, 1.19)	0.83 (0.43, 1.58)	0.59 (0.25, 1.24)	0.65 (0.30, 1.44)	0.60 (0.24, 1.32)	0.58 (0.26, 1.33)
**Anxiety[4]**								
IMPC	Ref	Ref	Ref	Ref	Ref	Ref	Ref	Ref
GMPC	0.84 (0.48, 1.46)	0.79 (0.45, 1.40)	0.75 (0.41, 1.34)	0.72 (0.39, 1.33)	0.70 (0.33, 1.39)	0.63 (0.28, 1.39)	0.62 (0.27, 1.30)	0.54 (0.23, 1.28)

Note: Bold values denote statistical significance at the $p < 0.05$ level; ITT: Intent to Treat; PC: Per Compliance; cRR: Crude Risk Ratio; aRR: Adjusted Risk Ratio; CI: Confidence Interval

[1]Adjusted for race/ethnicity, age at pregnancy onset, annual household income level, parity, gestational age at baseline survey completion, and baseline score category on outcome scale.
[2]Score of 14 or greater on the Perceived Stress Scale (PSS-10); this indicates moderate to severe perceived stress.
[3]Score of 10 or greater on the Patient Health Questionnaire depression scale (PHQ-8); this indicates clinically significant depressive symptoms.
[4]Score of 10 or greater on the Generalized Anxiety Disorder 7-item scale (GAD-7); this indicates clinically significant anxiety symptoms.

© 2024 Avalos LA et al. *JAMA Network Open.*

eTable 4. Associations Between GMPC and Postpartum Psychosocial (Sleep and Social Support) Outcomes

	ITT		PC1		PC5		PC70%	
**Outcome**	Mean Difference (95% CI)	Adjusted Mean Difference[1] (95% CI)	Mean Difference (95% CI)	Adjusted Mean Difference[1] (95% CI)	Mean Difference (95% CI)	Adjusted Mean Difference[1] (95% CI)	Mean Difference (95% CI)	Adjusted Mean Difference[1] (95% CI)
**Sleep Quality[2]**								
IMPC	Ref	Ref	Ref	Ref	Ref		Ref	Ref
GMPC	-0.60 (-1.3, 0.11)	-0.61 (-1.3, 0.12)	-0.58 (-1.30, 0.17)	-0.60 (-1.40, 0.17)	**-0.89 (-1.70, -0.07)**	-0.71 (-1.6, 0.16)	**-1.0 (-1.9, -0.18)**	-0.89 (-1.8, 0.03)
**Social Support[3]**								
IMPC	Ref	Ref	Ref	Ref	Ref	Ref	Ref	
GMPC	0.13 (-0.01, 0.26)	0.08 (-0.03, 0.19)	0.12 (-0.02, 0.26)	0.04 (-0.08, 0.15)	**0.20 (0.05, 0.34)**	0.12 (-0.01, 0.24)	**0.20 (0.04, 0.36)**	0.12 (-0.02, 0.26)

Note: Bold values denote statistical significance at the $p < 0.05$ level

[1]Adjusted for race/ethnicity, age at pregnancy onset, annual household income level, parity, gestational age at baseline survey completion, and baseline score on outcome scale.

[2]Pittsburgh Sleep Quality Index (PSQI) Score, Range: 0 (better) to 21 (worse)

[3]Medical Outcomes Study Social Support Survey (MOS-SSS) Score, Range: 1 (low)-5 (high)

© 2024 Avalos LA et al. *JAMA Network Open.*

eTable 5. Associations Between GMPC and Postpartum Behavioral (Breastfeeding and LARC Intention)

	ITT		PC1		PC5		PC70%	
**Outcome**	cRR (95% CI)	aRR[1] (95% CI)	cRR (95% CI)	aRR[1] (95% CI)	cRR (95% CI)	aRR[1] (95% CI)	cRR (95% CI)	aRR[1] (95% CI)
**Breastfeeding**								
IMPC	Ref	Ref	Ref	Ref	Ref	Ref	Ref	Ref
GMPC	1.02 (0.94, 1.09)	0.99 (0.93, 1.04)	1.04 (0.96, 1.11)	1.01 (0.95, 1.07)	1.05 (0.98, 1.13)	1.01 (0.95, 1.08)	**1.08 (1.01, 1.16)**	1.03 (0.96, 1.11)
**LARC Intention**								
IMPC	Ref	Ref	Ref	Ref	Ref	Ref	Ref	Ref
GMPC	0.92 (0.66, 1.27)	1.02 (0.71, 1.47)	0.92 (0.66, 1.29)	1.00 (0.69, 1.47)	1.00 (0.70, 1.43)	1.07 (0.71, 1.62)	1.14 (0.79, 1.64)	1.18 (0.77, 1.79)

Note: Bold values denote statistical significance at the p < 0.05 level; ITT: Intent to Treat; PC: Per Compliance; cRR: Crude Risk Ratio; aRR: Adjusted Risk Ratio; CI: Confidence Interval

Note: Bold values denote statistical significance at the p < 0.05 level

[1]Adjusted for race/ethnicity, age at pregnancy onset, annual household income level, parity, gestational age at baseline survey completion.

[2]Any self-reported breastfeeding since birth

[3]Self-reported intention to use a LARC (Long-Acting Reversible Contraceptive) method in the postpartum period

© 2024 Avalos LA et al. *JAMA Network Open.*

eTable 6. Associations Between GMPC and Perceived Quality of Prenatal Care

	ITT		PC1		PC5		PC70%	
**Outcome**	Mean Difference (95% CI)	Adjusted Mean Difference[1] (95% CI)	Mean Difference (95% CI)	Adjusted Mean Difference[1] (95% CI)	Mean Difference (95% CI)	Adjusted Mean Difference[1] (95% CI)	Mean Difference (95% CI)	Adjusted Mean Difference[1] (95% CI)
**Perceived Quality of Prenatal Care[2]**								
IMPC	Ref	Ref	Ref	Ref	Ref	Ref	Ref	Ref
GMPC	0.01 (-0.12, 0.13)	0.01 (-0.12, 0.15)	0.01 (-0.11, 0.14)	0.02 (-0.12, 0.16)	0.14 (0.00, 0.28)	**0.16 (0.01, 0.31)**	**0.20 (0.05, 0.35**)	**0.22 (0.05, 0.38)**

[1]Adjusted for race/ethnicity, age at pregnancy onset, annual household income level, parity, gestational age at baseline survey completion.
[2]Quality of Prenatal Care Questionnaire (QPCQ) Total 46-item mean score, Range: 1 (worse)-5 (better)

© 2024 Avalos LA et al. *JAMA Network Open.*

eTable 7. Associations of GMPC with Postpartum Sleep, Social Support, and Perceived Quality of Prenatal Care Subscales

	ITT		PC1		PC5		PC70%	
	Mean (SD)	Adjusted mean difference[1] (95% CI)	Mean (SD)	Adjusted mean difference[1] (95% CI)	Mean (SD)	Adjusted mean difference[1] (95% CI)	Mean (SD)	Adjusted mean difference[1] (95% CI)
**Sleep Quality Component Scores[2]**								
**Duration of sleep**								
IMPC	1.53 (0.74)	Ref	1.54 (0.74)	Ref	1.51 (0.73)	Ref	1.53 (0.72)	Ref
GMPC	1.45 (0.77)	**-0.19 (-0.35, -0.03)**	1.46 (0.77)	**-0.20 (-0.37, -0.03)**	1.50 (0.75)	-0.08 (-0.28, 0.11)	1.53 (0.75)	-0.08 (-0.29, 0.13)
**Sleep Disturbance**								
IMPC	0.97 (1.00)	Ref	0.95 (1.00)	Ref	0.95 (1.01)	Ref	0.97 (1.02)	Ref
GMPC	0.83 (0.94)	-0.08 (-0.27, 0.11)	0.82 (0.95)	-0.04 (-0.25, 0.16)	0.71 (0.87)	-0.10 (-0.34, 0.13)	0.68 (0.88)	-0.15 (-0.40, 0.11)
**Sleep Latency**								
IMPC	1.35 (1.06)	Ref	1.36 (1.06)	Ref	1.39 (1.06)	Ref	1.43 (1.06)	Ref
GMPC	1.24 (1.03)	-0.14 (-0.36, 0.08)	1.23 (1.03)	-0.17 (-0.40, 0.06)	1.15 (0.92)	**-0.27 (-0.54, -0.01)**	1.18 (0.94)	**-0.30 (-0.58, -0.02)**
**Day Dysfunction due to Sleepiness**								
IMPC	1.80 (1.12)	Ref	1.79 (1.13)	Ref	1.81 (1.14)	Ref	1.84 (1.14)	Ref
GMPC	1.72 (1.16)	-0.15 (-0.40, 0.09)	1.76 (1.16)	-0.12 (-0.37, 0.14)	1.83 (1.09)	-0.03 (-0.33, 0.27)	1.86 (1.10)	-0.04 (-0.36, 0.27)
**Sleep Efficiency**								
IMPC	1.12 (0.50)	Ref	1.12 (0.50)	Ref	1.14 (0.50)	Ref	1.14 (0.51)	Ref
GMPC	1.13 (0.55)	0.02 (-0.09, 0.12)	1.13 (0.55)	0.01 (-0.10, 0.12)	1.08 (0.44)	-0.04 (-0.16, 0.08)	1.07 (0.41)	-0.03 (-0.16, 0.09)
**Overall Sleep Quality**								
IMPC	0.34 (0.81)	Ref	0.34 (0.81)	Ref	0.35 (0.82)	Ref	0.35 (0.83)	Ref
GMPC	0.21 (0.70)	-0.04 (-0.20, 0.12)	0.22 (0.71)	-0.03 (-0.20, 0.13)	0.16 (0.60)	-0.06 (-0.24, 0.13)	0.12 (0.54)	-0.08 (-0.27, 0.12)
**Needs Medications to Sleep**								
IMPC	0.84 (0.82)	Ref	0.85 (0.82)	Ref	0.85 (0.81)	Ref	0.86 (0.82)	Ref

© 2024 Avalos LA et al. *JAMA Network Open.*

GMPC	0.77 (0.84)	-0.04 (-0.21, 0.14)	0.75 (0.80)	-0.08 (-0.26, 0.10)	0.70 (0.77)	-0.13 (-0.33, 0.07)	0.66 (0.76)	-0.20 (-0.42, 0.01)
**Social Support Subscales[3]**								
**Tangible support**								
IMPC	4.39 (0.83)	Ref	4.39 (0.83)	Ref	4.42 (0.84)	Ref	4.41 (0.83)	Ref
GMPC	4.53 (0.71)	0.05 (-0.09, 0.19)	4.51 (0.74)	0.00 (-0.15, 0.15)	4.64 (0.56)	0.11 (-0.06, 0.28)	4.62 (0.57)	0.09 (-0.09, 0.27)
**Positive social interaction**								
IMPC	4.36 (0.87)	Ref	4.35 (0.88)	Ref	4.38 (0.87)	Ref	4.36 (0.88)	Ref
GMPC	4.56 (0.75)	0.14 (0.01, 0.29)	4.56 (0.75)	0.12 (-0.03, 0.27)	4.67 (0.59)	**0.17 (0.01, 0.33)**	4.65 (0.61)	0.16 (-0.02, 0.33)
**Emotional/Informational Support**								
IMPC	4.24 (0.75)	Ref	4.25 (0.74)	Ref	4.26 (0.75)	Ref	4.24 (0.75)	Ref
GMPC	4.33 (0.79)	0.08 (-0.05, 0.22)	4.32 (0.80)	0.04 (-0.10, 0.18)	4.40 (0.71)	0.12 (-0.04, 0.28)	4.38 (0.73)	0.15 (-0.02, 0.32)
**Affectionate Support**								
IMPC	4.56 (0.75)	Ref	4.55 (0.76)	Ref	4.57 (0.74)	Ref	4.56 (0.75)	Ref
GMPC	4.68 (0.67)	0.05 (-0.07, 0.17)	4.68 (0.69)	0.02 (-0.11, 0.1)	4.78 (0.54)	0.10 (-0.04, 0.24)	4.75 (0.58)	0.09 (-0.06, 0.24)
**Quality of Prenatal Care Subscales[4]**								
**Information Sharing**								
IMPC	4.37 (0.63)	Ref	4.37 (0.63)	Ref	4.36 (0.64)	Ref	4.34 (0.64)	Ref
GMPC	4.33 (0.62)	-0.05 (-0.18, 0.09)	4.33 (0.63)	-0.05 (-0.19, 0.09)	4.45 (0.54)	0.09 (-0.07, 0.24)	4.48 (0.52)	0.14 (-0.02, 0.31)
**Anticipatory Guidance**								
IMPC	3.95 (0.76)	Ref	3.96 (0.76)	Ref	3.93 (0.76)	Ref	3.90 (0.76)	Ref
GMPC	4.05 (0.72)	0.14 (-0.02, 0.30)	4.07 (0.73)	0.17 (0.01, 0.33)	4.18 (0.63)	**0.31 (0.13, 0.49)**	4.24 (0.62)	**0.38 (0.19, 0.57)**
**Sufficient Time**								
IMPC	3.97 (0.85)	Ref	3.99 (0.84)	Ref	3.97 (0.86)	Ref	3.96 (0.86)	Ref
GMPC	3.98 (0.77)	0.02 (-0.16, 0.19)	4.01 (0.74)	0.04 (-0.14, 0.22)	4.07 (0.75)	0.16 (-0.05, 0.37)	4.15 (0.69)	**0.24 (0.02, 0.47)**

© 2024 Avalos LA et al. *JAMA Network Open.*

**Approachability**								
IMPC	4.19 (0.92)	Ref	4.19 (0.93)	Ref	4.22 (0.88)	Ref	4.21 (0.88)	Ref
GMPC	4.13 (0.88)	-0.07 (-0.27, 0.12)	4.19 (0.82)	-0.01 (-0.22, 0.19)	4.31 (0.77)	0.07 (-0.15, 0.29)	4.32 (0.78)	0.08 (-0.15, 0.31)
**Availability**								
IMPC	4.13 (0.77)	Ref	4.14 (0.77)	Ref	4.12 (0.79)	Ref	4.10 (0.78)	Ref
GMPC	4.17 (0.69)	0.07 (-0.09, 0.23)	4.16 (0.70)	0.05 (-0.11, 0.22)	4.29 (0.61)	**0.22 (0.03, 0.41)**	4.30 (0.61)	**0.25 (0.06, 0.45)**
**Support and Respect**								
IMPC	4.37 (0.68)	Ref	4.38 (0.68)	Ref	4.37 (0.69)	Ref	4.35 (0.69)	Ref
GMPC	4.32 (0.63)	-0.06 (-0.21, 0.08)	4.34 (0.63)	-0.05 (-0.21, 0.10)	4.46 (0.54)	0.08 (-0.09, 0.25)	4.49 (0.51)	0.14 (-0.04, 0.31)

Note: Bold values indicate statistical significance at the $p<0.05$ level.

[1]Adjusted for race/ethnicity, age at pregnancy onset, annual household income level, parity, gestational age at baseline survey completion, and baseline score on outcome scale.

[2]Pittsburgh Sleep Quality Index (PSQI) component scores postpartum (Range: 0 (Better) – 3 (Worse))

[3]Medical Outcomes Study Social Support Survey (MOS-SSS) subscale scores postpartum (Range: 1 (low)-5 (high))

[4]Quality of Prenatal Care Questionnaire (QPCQ) subscale scores (Range: 1 (worse)-5(better))

© 2024 Avalos LA et al. *JAMA Network Open.*

eMethods. Additional Details on Identification and Recruitment of the Study Sample.

Identification and recruitment of GMPC participants

Potential GMPC participants were identified from the EHRs using the unique Centering procedure code for a future appointment and sent recruitment emails. Of the 551 GMPC participants identified, 212 enrolled, 9 (2%) were ineligible, 49 (8%) refused, and 281 (51%) were unable to contact. Reasons for ineligibility included: did not speak English (n=6), residing outside the KPNC service area (n=2), and disenrollment from GMPC (n=1). Reasons for refusal included: no interest (n=14), lack of time (n=1), stress (n=1), and non-completion or partial completion of the baseline survey after agreeing at the recruitment call (n=33).

Of the 212 GMPC participants who enrolled in the study, 5 experienced a pregnancy loss (2%) resulting in 207 participants with a live birth. Of these 207, 198 (96%) completed the follow-up survey (including 3 partial completes), 8 (4%) were lost to follow-up and 1 (<1%) refused the follow-up survey (Figure 1).

Identification and recruitment of IMPC participants

A total of 2,955 IMPC controls were identified from clinics that did not offer GMPC through frequency matching based on gestational age, race/ethnicity, Medicaid enrollment, and maternal age for the 212 enrolled Centering participants. Up to 15 frequency-matched controls were identified for each GMPC participant and sent a recruitment email. The first individual for each GMPC participant who responded that they were interested in the study received a follow-up phone call to assess eligibility. Potential controls were asked if they would have participated in GMPC if it was available at their clinic and offered to them. Those who responded "yes" were recruited. The recruiter also followed up with individuals who were sent an email until a control was enrolled for each GMPC participant. If more controls were needed, up to 15 more were

© 2024 Avalos LA et al. *JAMA Network Open.*

identified from the EHRs. The recruitment emails included consent information embedded in the body and the link to the survey.

Of the 2,955 IMPC controls identified, 212 enrolled in the study, 91 (2%) refused, 275 were unable to contact (9%), and 14 (<1%) were ineligible. A match had been found prior to needing to assess eligibility for 2,187 individuals and prior to 174 responding that they were interested in the study.

Of the 212 enrolled IMPC participants 10 (4.7%) experienced a pregnancy loss resulting in 202 individuals with a live birth. One multiple gestation pregnancy identified after study enrollment was excluded from the analysis, resulting in 201 participants with a singleton live birth. Of these 201 participants, 192 (95.0%) completed a postpartum follow-up survey (including 3 partial completes), 7 (3.5%) were lost to follow-up, and 2 (1%) refused the follow-up survey (Figure 1).

© 2024 Avalos LA et al. *JAMA Network Open.*

eAppendix. The Quality of Prenatal Care Questionnaire Subscales

Subscale	Items in each subscale
Information Sharing (9 items)	I was given adequate information about prenatal tests and procedures.<br>I was given honest answers to my questions.<br>Everyone involved in my prenatal care received important information about me.<br>I was screened adequately for potential problems with my pregnancy.<br>The results of the tests were explained to me in a way that I could understand.<br>My prenatal care provider(s) gave straightforward answers to my questions.<br>My prenatal care providers gave me enough information to make decisions for myself.<br>My prenatal care provider kept my information confidential.<br>I fully understood the reasons for blood work and other tests my prenatal care provider(s) ordered for me.
Anticipatory Guidance (11 items)	My prenatal care provider(s) gave me options for my birth experience.<br>I was given enough information to meet the needs about breastfeeding.<br>My prenatal care provider(s) prepared me for my birth experience.<br>My prenatal care provider(s) spent time talking with me about my expectations for labor and delivery.<br>I was given enough information about the safety of moderate exercise during pregnancy.<br>I received adequate information about my diet during pregnancy.<br>My prenatal care provider(s) was interested in how my pregnancy was affecting my life.<br>I was linked to programs in the community that were helpful to me.<br>I received adequate information about alcohol use during pregnancy.<br>I was given adequate information about depression during pregnancy.<br>My prenatal care provider(s) took time to ask about things that were important to me
Sufficient Time (5 items)	I had as much time with my prenatal care provider(s) as I needed.<br>My prenatal care provider(s) was rushed.<br>My prenatal care provider(s) always had time to answer my questions.<br>My prenatal care provider(s) made time for me to talk.<br>My prenatal care provider(s) took time to listen.
Approachability (4 items)	My prenatal care provider(s) was abrupt with me.<br>I was rushed during my prenatal care visits.<br>My prenatal care provider(s) made me feel like I was wasting their time.<br>I was afraid to ask my prenatal care provider(s) questions.

© 2024 Avalos LA et al. *JAMA Network Open.*

Availability (5 items)	I knew how to get in touch with my prenatal care provider(s).<br>Someone in my prenatal care provider(s)'s office always returned my calls.<br>My prenatal care provider(s) was available when I had questions or concerns.<br>I could always reach someone in the office/clinic if I needed something.<br>I could reach my prenatal care provider(s) by phone when necessary.
Support and Respect (12 items)	My prenatal care provider(s) respected me.<br>My prenatal care provider(s) respected my knowledge and experience.<br>My decisions were respected by my prenatal care provider(s).<br>My prenatal care provider(s) was patient.<br>I was supported by my prenatal care provider(s) in doing what I felt was right for me.<br>My prenatal care provider(s) supported me.<br>My prenatal care provider(s) paid close attention when I was speaking.<br>My concerns were taken seriously.<br>I was in control of the decisions being made about my prenatal care.<br>My prenatal care provider(s) supported my decisions.<br>I was at ease with my prenatal care provider(s).<br>My values and beliefs were respected by my prenatal care provider(s).

© 2024 Avalos LA et al. *JAMA Network Open.*
