# Supplementary material for: Group Multimodal Prenatal Care and Postpartum Outcomes
Source: JAMA Netw Open. 2024 May 21;7(5):e2412280. doi: 10.1001/jamanetworkopen.2024.12280 (PMC11109777; doi:10.1001/jamanetworkopen.2024.12280)
Supplement: Supplement 2. — Data Sharing Statement [file jamanetwopen-e2412280-s002.pdf]

Data Sharing Statement

Avalos. Group Multimodal Prenatal Care and Postpartum Outcomes. *JAMA Netw Open*.
Published May 21, 2024. doi:10.1001/jamanetworkopen.2024.12280

Data
Data available: No
